# Oral tolerogenic vaccine combined with gastrin restores immune tolerance and beta-cell function in NOD mice with Type 1 diabetes

**DOI:** 10.3389/fimmu.2025.1740385

**Published:** 2026-01-19

**Authors:** Jacob Cobb, Jeffrey Rawson, Nelson Gonzalez, Fouad Kandeel, Mohamed I. Husseiny

**Affiliations:** Department of Translational Research & Cellular Therapeutics, Arthur Riggs Diabetes & Metabolism Research Institute, Beckman Research Institute, City of Hope National Medical Center, Duarte, CA, United States

**Keywords:** combination therapy, gastrin, immune tolerance, oral *Salmonella*-based vaccine, regulatory T-cells, type 1 diabetes

## Abstract

**Background and objective:**

Type 1 diabetes (T1D) results from autoimmune destruction of pancreatic β-cells. Current therapies fail to address the multiple mechanisms driving disease progression. We developed an oral *Salmonella*-based vaccine that partially prevented and reversed autoimmune diabetes in mice. Gastrin, an intestinal hormone, has been reported to have anti-inflammatory and β-cell-protective effects. We hypothesized that combining the vaccine with a gastrin analogue (GAST-17) could enhance therapeutic efficacy.

**Methods:**

Female non-obese diabetic (NOD) mice were treated with the oral vaccine, GAST-17, or their combination. Blood glucose levels, islet histology, immune cell infiltration, cytokine profiles, and regulatory T cell populations were assessed. Functional assays included antigen-specific stimulation, adoptive transfer, and analysis of immunoregulatory gene expression.

**Results:**

Combination therapy demonstrated superior efficacy in both diabetes reversal and prevention. In reversal studies, diabetes remission was achieved in 80% of mice receiving the combination therapy, compared with 63% in the vaccine-only group and 5% in the GAST-17-only group. In prevention studies, diabetes onset was prevented in 80% of mice receiving the combination therapy, compared with 70% in the vaccine-only group and 30% in the GAST-17-only group. Therapeutic effects were associated with increased antigen-specific regulatory T-cells, reduced islet-infiltrating lymphocytes, preserved insulin-positive islet area and β-cell mass, and modulation of cytokine profiles, including elevated IL-10 and TGF-β and reduced IFN-γ, GM-CSF, IL-1α, and IL-12. Upregulation of immune checkpoint molecules (CTLA-4 and PD-L1) and immunoregulatory mediators (AhR, IDO, and IL-27) was observed, suggesting a potential contribution to immune homeostasis.

**Conclusions:**

The combination of the oral *Salmonella*-based vaccine and GAST-17 improved glycemic control in NOD mice and was strongly associated with β-cell preservation and immune regulation. This dual-acting strategy, integrating immune modulation with β-cell preservation, may offer durable therapy in autoimmune diabetes and could have potential for future clinical translation.

## Introduction

Type 1 diabetes (T1D) is a complex autoimmune disease characterized by the progressive destruction of pancreatic β-cells, leading to insulin deficiency and impaired glycaemic control (1–4). Insulin is a key autoantigen in T1D, and its targeted immune response contributes to β-cells loss and systemic metabolic dysregulation (5–8). Due to the multifactorial pathophysiology of T1D, monotherapy is often insufficient to achieve durable glycemic control or prevent disease progression and the related complications. Exogenous insulin is essential for survival but it frequently does not maintain glucose within a physiological range, contributing to recurrent hyper- and hypoglycemia, and neural, renal, and retinal pathology, which impair the quality of life and reduce longevity (9–11).

Restoration immune tolerance to autoantigens is therapeutic strategy being pursued for many diseases including T1D. Autoantigen-based therapies increased antigen-specific regulatory T-cells (Tregs), promoted immune homeostasis, and reversed hyperglycemia in animals (12–15). Building on this, we developed an oral *Salmonella*-based vaccine delivering the T1D autoantigen preproinsulin (PPI) in combination with the immunoregulatory cytokines TGF-β and IL-10 (16, 17). When administered alongside low-dose anti-CD3 monoclonal antibody, the vaccine prevented autoimmune diabetes in 70% of non-obese diabetic (NOD) mice and reversed newly established diabetes in 50% of animals (17–19). These therapeutic effects correlated with increased numbers of antigen-specific Tregs and type 1 regulatory T-cells (Tr1) in the spleen, mesenteric lymph nodes (MLNs), pancreatic lymph nodes (PLNs), and pancreatic islets, and this was accompanied by reduced islet-infiltrating lymphocytes and increased β-cell mass (17–20). Importantly, vaccinated mice retained an immune response against *Salmonella* clearing the bacteria within four weeks (20–24) while dendritic cells (DCs) from vaccinated animals were tolerogenic preventing diabetes upon transfer into naïve T1D -prone mice (20, 25). An advantage of this delivery system is its dosing simplicity. Only two oral administrations were sufficient to induce immune modulation (17), compared with the intensive dosing required for the *Lactococcus* vaccine (five times weekly for six weeks) (26, 27). Furthermore, the safety of the vector is supported by use of an FDA-approved oral *Salmonella* vaccine (Vivotif), with more than 150 million doses administered and, as of yet, no major adverse events reported (28, 29).

However, durable reversal of T1D requires immune modulation and support of β-cells. Gastrin, a peptide hormone produced by gastric antral, duodenal, and pancreatic G cells, acts as β-cell restorative. Interestingly, gastrin stimulated β-cell neogenesis from exocrine pancreatic tissue, increased islet mass, and improved glucose tolerance in mice (30–33). In individuals with T1D, gastrin is expressed in insulin- and somatostatin-producing islet cells, suggesting a possible role in promoting β-cell health and limiting inflammation (34). Gastrin treatment decreased islet inflammation, expanded β-cell mass, restored glycemic control, and enhanced survival and function of human islets transplanted into rodents (35–39). These benefits extended even to human islets from individuals with poor glycemic control. Here, gastrin increased expression of endocrine gene transcripts, including insulin, glucagon, somatostatin, and β-cell identity factors such as PDX1, MNX1, MAFA, NKX6.1, and NKX6.2 (40).

Effective therapy for T1D should suppress autoreactive T-cells, restore Treg function, establish immune tolerance, and support β-cell health (41). In this study, we evaluated the therapeutic potential of combining an oral *Salmonella*-based vaccine and GAST-17, a gastrin analogue, in diabetic NOD mice. Combined therapy reduced hyperglycemia in both preventive and curative models of autoimmune diabetes. The combination treatment promoted expansion of antigen-specific Tregs, suppressed islet inflammation, and enhanced β-cell survival, growth, and function more than either treatment alone.

## Materials and methods

### *Salmonella* vaccine preparation

Live attenuated *Salmonella msbB* mutants were engineered to express of proinsulin (PI) and the immunomodulatory cytokines IL-10 and TGF-β (17–19, 25). The final vaccine formulation included *Salmonella* carrying PI+IL-10, PI+TGF-β, and was administered in combination with anti-CD3 monoclonal antibody (17–19, 25).

### Mice

Female NOD/ShiLtJ and NSG (NOD.*Cg-Prkdc^scid^ Il2rg^tm1Wjl^*/SzJ) mice aged 7–8 weeks were obtained from Jackson Laboratory (Bar Harbor, ME, USA) and maintained in a pathogen-free, AAALAC-accredited facility. All procedures were approved by the Institutional Animal Care and Use Committee of City of Hope (IACUC# 18017).

### Animal vaccination

Groups of NOD mice were randomly assigned to receive:

- Vehicle control (oral 200 µl 5% sodium bicarbonate).
- GAST-17 (600 µg/kg intraperitoneally (i.p.) once daily, starting on the day of vaccination) (42).
- Vaccine (two oral gavage doses one week apart; 200 µl in 5% sodium bicarbonate) (22, 23, 43, 44).
- Vaccine + GAST-17 (combined as above).

Anti-CD3 antibody (2.5 µg i.p.) was administered for five consecutive days starting one day before vaccination (days -1 to 3) (17–20, 25).

*Prevention model*: Normoglycemic NOD mice (blood glucose < 200 mg/dL) were treated at 8 weeks of age and monitored for diabetes onset for 90 days.

*Reversal model*: Diabetic NOD mice (blood glucose > 200 mg/dL) were treated and monitored for reversal of hyperglycemia.

Blood glucose values were measured twice a week. Mice were deemed diabetic if blood glucose values were ≥ 200 mg/dL on two consecutive measurements (17–20, 25).

### Histology and immunofluorescence

Pancreatic tissue was fixed, paraffin-embedded, and stained with hematoxylin and eosin. Islet infiltration was evaluated in a blinded manner across 4 non-adjacent sections per pancreas (each 5 µm thick), separated by at least 150 µm, using light microscopy at 20x magnification. Insulitis was scored as follows (0 = no insulitis; 1 = peri-insulitis; 2 = mild insulitis with <50% islet area affected; 3 = invasive insulitis with >50% islet area affected, 4 = invasive insulitis with 100% islet area affected). Islets obtained from 5 to 10 mice per group were analyzed, with 30 and 132 islets evaluated per mouse (16, 17, 45).

Immunofluorescence staining was performed using a guinea pig anti-insulin antibody (Invitrogen) and a donkey anti-guinea pig Alexa Fluor 488-conjugated secondary antibody (Jackson ImmunoResearch). Nuclei were counterstained with DAPI. Images were acquired using a ZEISS LSM700 microscope and analyzed with ZEN-lite software. The β-cell fraction was calculated as the percentage of the area of insulin-positive cells divided by the total islet area. Islets were obtained from the pancreatic tissues of 10 mice per group. For each mouse, 61 to 71 islets were evaluated across 4 slides collected from different section levels (each 5 µm thick), with at least 150 µm separating each level. Data were analyzed using QuPath software (17, 45).

### Flow cytometric analysis

Single-cell suspensions from spleens were prepared following collagenase D digestion. Cells were stained with antibodies against CD4, CD8a, CD25, and FOXP3 to identify Tregs, as well as CD49b, LAG-3 to identify type 1 regulatory T cells. Matching isotype controls (BioLegend, San Diego, CA, USA) were included, UltraComp eBeads Compensation Beads (Invitrogen) were used to set compensation controls. Dead cells were excluded using Fixable Blue Dead Cell Stain (Invitrogen). Samples were acquired on a BD LSRFortessa flow cytometer, and data were analyzed using FlowJo software (17–20, 25).

### *In vitro* antigen-specific responsiveness

To assess antigen specificity, 5 x 10^5^ splenocytes were isolated and cultured in a 96-well plate for 72 hours with 10 µg/ml insulin B_9–23_ peptide or OVA_323-339_, a non-specific peptide (17). A Mouse Quantikine ELISA Kit (R&D Systems) was used to quantify IL-10, TGF-β, IFN-γ, and TNFα levels in the conditioned media of the treated splenocytes.

### *In vivo* suppressive activity of Tregs after adoptive transfer

Splenocytes from three diabetic NOD mice were isolated, pooled, and transferred into ten immunodeficient NSG mice (18, 20). Four weeks after vaccination, CD4^+^ CD25^+^ Tregs were isolated from five NOD mice treated with vehicle, vaccine, GAST-17, or both. Ten NSG mice received either 1 x 10^6^ diabetogenic cells alone or with 5 x 10^5^ Tregs from each treatment group. GAST-17 (600 µg/kg) was given i.p. once daily to mice receiving Tregs from the GAST-17 or GAST-17 and vaccine treatment groups, starting on the day of cell transfer. Blood glucose levels were measured twice weekly in NSG mice (18, 20).

### Cytokine measurement

Mouse serum cytokine levels were quantified using the MILLIPLEX Mouse High Sensitivity T Cell Panel - Immunology Multiplex Assay (Millipore, Sigma) (16, 20).

### Gene expression

Four weeks after vaccination, splenocytes and CD4^+^ CD25^+^ Tregs and CD4^+^ CD25^–^ cells were isolated from treated mice. RNA was extracted, converted to cDNA, and analyzed by qPCR analysis using Taq-Man assays (Applied Biosystem) to measure gene expression (20). mRNA levels of PDL-1, CTLA-4, AhR, IDO, and IL-27 were normalized to TATA box binding protein (Tbp) expression (20).

### Statistical analysis

Statistical analyses were performed using GraphPad Prism 10. Kaplan-Meier plots were employed to evaluate the incidence and reversal of diabetes across treatment groups, with statistical significance assessed using the Mantel-Cox log-rank test. Data distribution and normality were assessed using the Shapiro-Wilk test. Parametric tests were applied when the data met statistical assumptions, particularly normality (p > 0.05). In contrast, non−parametric tests were used when the assumptions were not met, and the data were not normally distributed (p < 0.05). Two-way ANOVA (Bonferroni’s multiple comparison test) was used to assess immune cell infiltration in islets and cytokine secretion following peptide stimulation. Dunn’s multiple comparisons test following the Kruskal-Wallis test was employed to compare the percentage of insulin-positive β-cells per islet across treatment groups. Serum cytokine levels among treatment groups were analyzed using one-way ANOVA for normally distributed data, followed by Fisher’s LSD multiple comparisons test. For non-normally distributed data, the Kruskal-Wallis test was used, followed by Dunn’s multiple comparisons test. One-way ANOVA followed by Tukey’s multiple comparisons test was used to compare Treg and Tr1 cell populations. Differences in cytokine levels and gene expression fold changes between treatment groups were analyzed using the unpaired Welch’s t-test for parametric data and using the Mann Whitney test for non-parametric data. A p-value < 0.05 was considered statistically significant.

## Results

### Combination therapy prevents diabetes onset and reverses hyperglycemia in NOD mice

**Figure 1:**
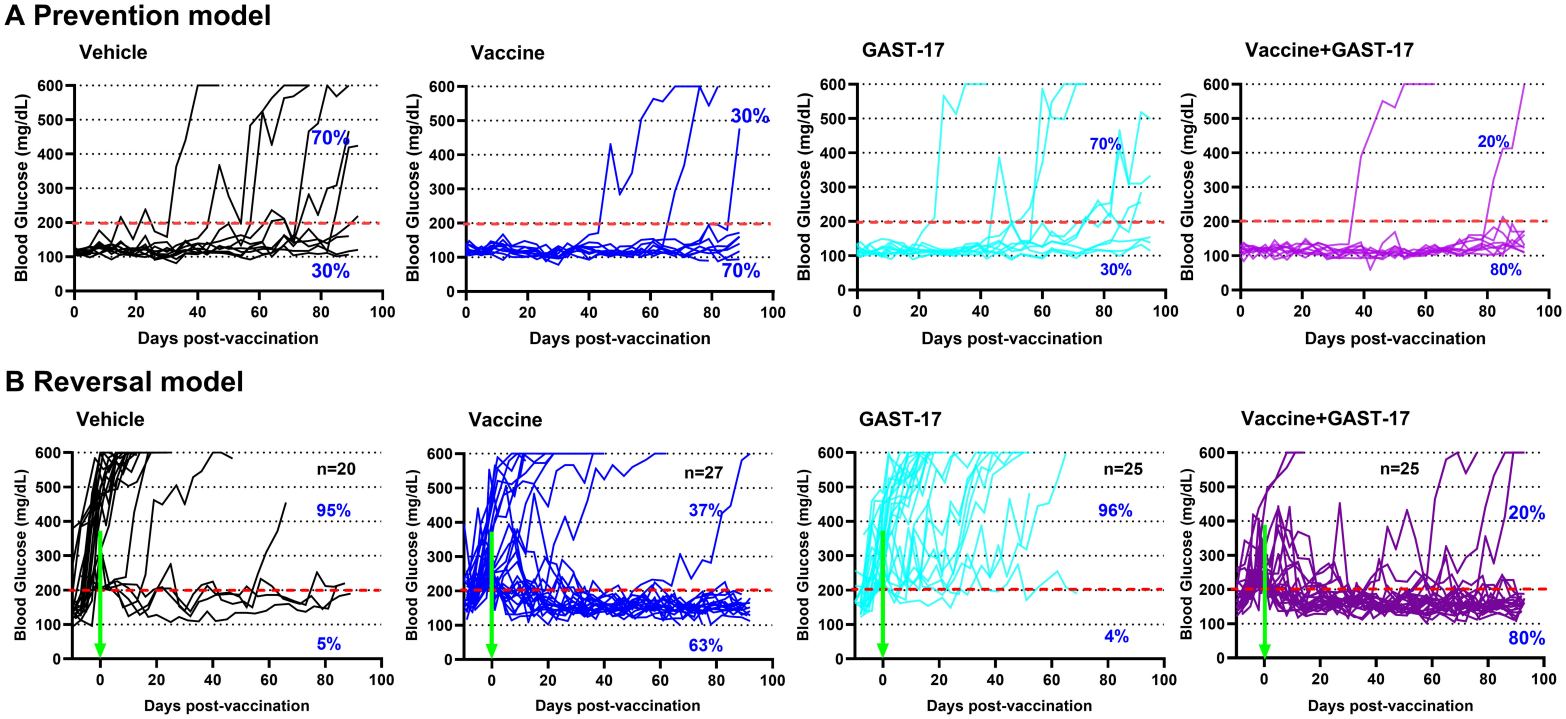
Combined therapy reduces hyperglycemia and reverses diabetes in NOD mice. Female NOD mice received 2 oral doses of the *Salmonella*-based vaccine (one week apart) and five-days i.p. of anti-CD3 mAb, followed by daily GAST-17 administered i.p. starting on the first day of vaccination. **(A)** Prevention model: blood glucose levels were monitored for 3 months in mice treated with, vehicle, vaccine, GAST-17, or the combination of vaccine and GAST-17 (n=10 per group). The experiment was repeated 3 times with similar results. **(B)** Reversal model: blood glucose levels were monitored for 3 months in diabetic mice treated with vehicle (n=20), vaccine (n=27), GAST-17 (n=25), or the combination of vaccine and GAST-17 (n=25). The red dotted line indicates a blood glucose level of 200 mg/dL, and green arrows mark the initiation of vaccination.

In the prevention model, 80% of normoglycemic NOD mice treated with the combination of oral vaccine and GAST-17 remained diabetes-free for 90 days, compared with 30% of mice receiving GAST-17 alone (**Figure 1A**, **Supplementary Figure 1A**).

In the reversal model (**Figure 1B**, **Supplementary Figure 1A**), diabetes was reversed in 80% of NOD mice treated with the combination therapy and in 63% of those receiving the vaccine alone. Both groups achieved and maintained normoglycemia for three months post-treatment (**Figure 1B**). In contrast, 95% of vehicle-treated and 96% of GAST-17-treated mice remained hyperglycemic (**Figure 1B**).

### Combination therapy preserves β-cells and limits immune cell infiltration of islets of NOD mice

**Figure 2:**
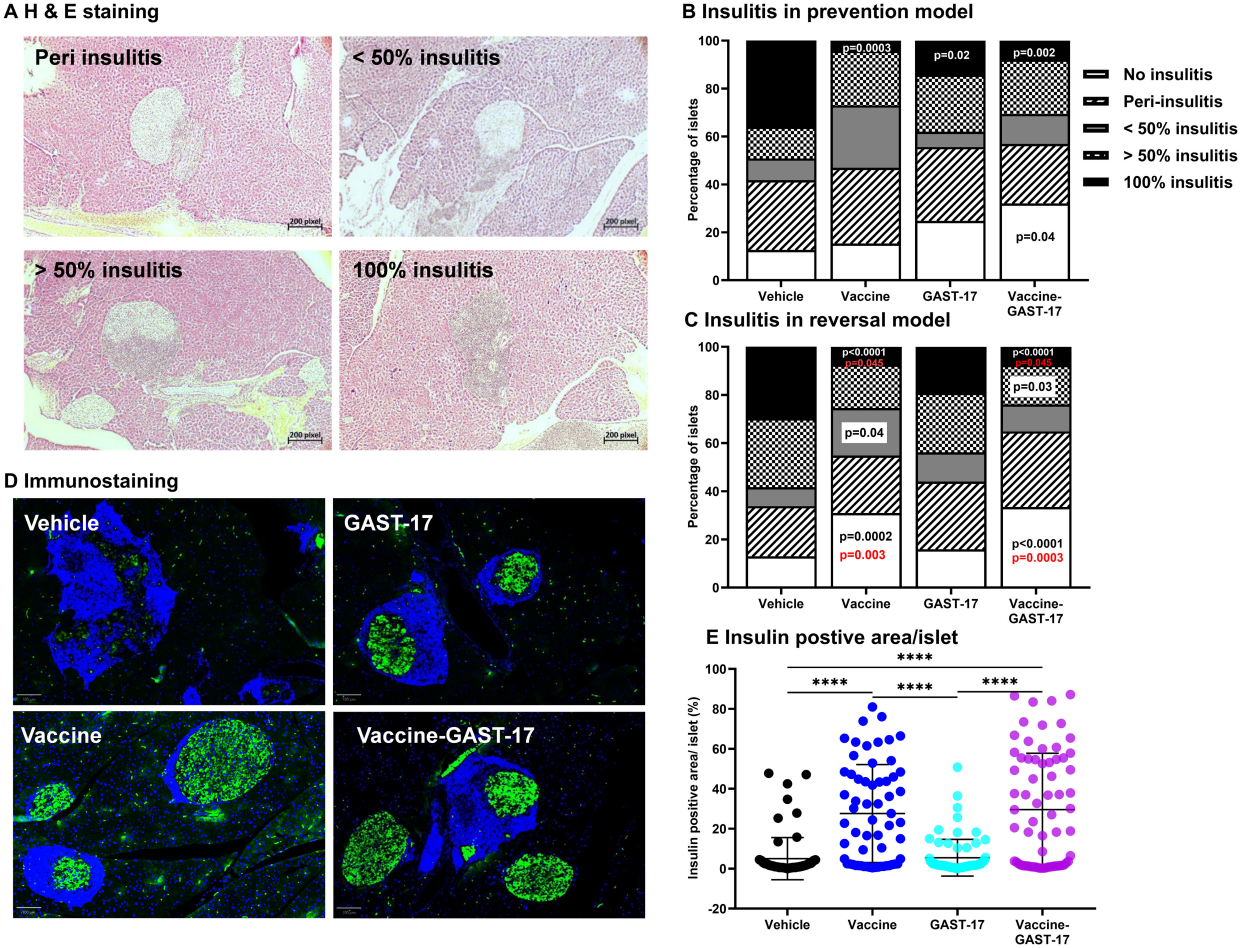
Combined therapy mitigates insulitis and preserves insulin-positive cells. **(A)** Pancreatic paraffin sections were stained with H&E. Islets were examined, counted, and graded for islet immune cell infiltration in a blinded manner in samples from the prevention **(B)** and reversal **(C)** model. Statistical significance was determined by Bonferroni’s multiple comparisons test following two-way ANOVA. Comparisons versus the vehicle group are indicated by black or white p-values. Comparisons versus GAST-17 alone are indicated by red p-values. **(D)** Representative pancreatic sections were immunostained for insulin (green) and nuclei (blue). **(E)** β-cells content was quantified as the percentage of insulin-positive cells per islet. Data is represented as mean ± SD from 8 to 10 mice per group. Statistical significance was assessed using Dunn’s multiple comparisons test following the non-parametric Kruskal-Wallis test (****p < 0.0001).

Histological analysis confirmed marked T-cell infiltration in pancreatic islets of vehicle-treated NOD mice (**Figure 2**, **Supplementary Figure 2**), both pre-diabetic (**Figure 2A**) and diabetic (**Figure 2C**), consistent with typical T1D pathology. Treatment with either the vaccine or GAST-17 alone significantly reduced islet immune infiltration compared to the vehicle group (**Figure 2A-C**). In the prevention model, the combined vaccine and GAST-17 therapy further decreased lymphocyte infiltration and preserved islet insulin content compared with vehicle-treated mice (**Figure 2B**; two-way ANOVA, p = 0.04, and p = 0.002). Vaccine alone also decreased islet inflammation compared with vehicle (**Figure 2B**; p = 0.0003). In the reversal model, diabetic mice receiving the combined treatment showed significantly reduced immune cell infiltration compared with mice treated with vehicle or GAST-17 alone (**Figure 2C**; two-way ANOVA, p < 0.0001, p = 0.03, and p < 0.0001; or p = 0.0003, respectively). Similarly, mice treated with the vaccine alone exhibited decreased islet inflammation compared with vehicle or GAST-17 alone (**Figure 2C**; p = 0.0002, p = 0.04, and p < 0.0001; or p = 0.003, respectively). Islets from mice treated with the vaccine or the combined vaccine and GAST-17 showed a significantly higher percentages of insulin-positive cells compared to those treated with vehicle or GAST-17 alone (**Figures 2D-E**; one-way ANOVA, p < 0.0001).

### Combination therapy decreases inflammatory cytokine levels

**Figure 3:**
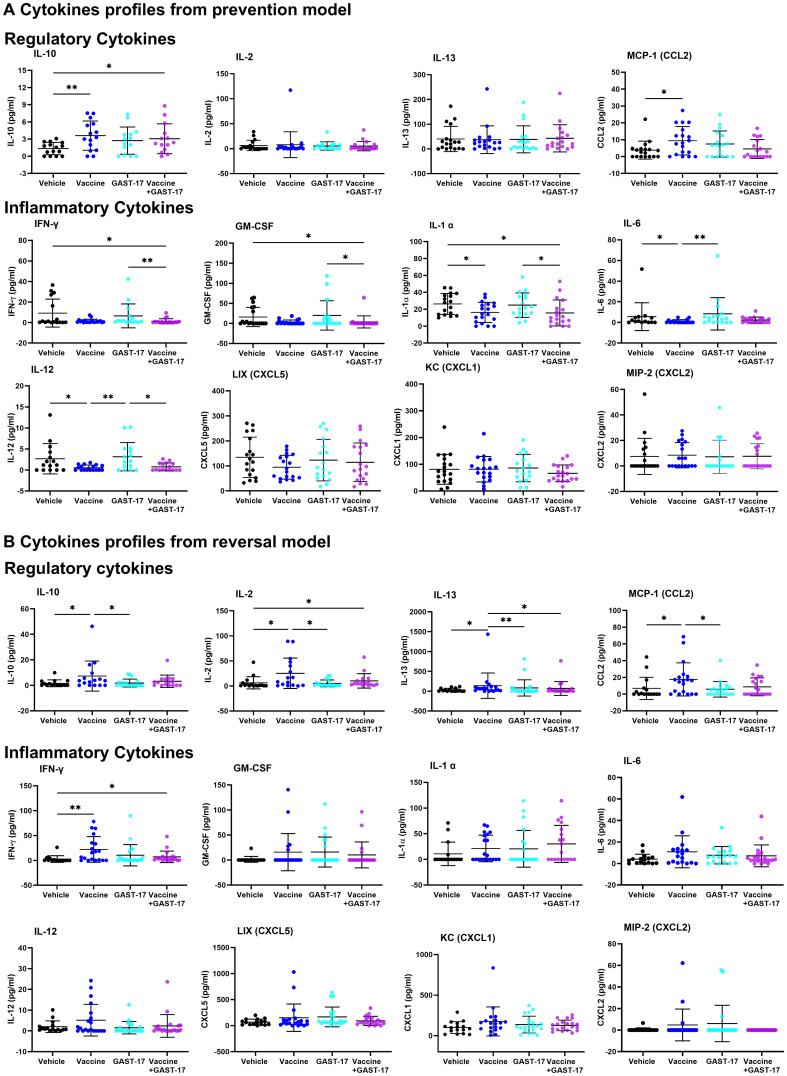
Effect of combination therapy on circulating regulatory and inflammatory cytokines. Serum cytokine concentrations were measured using a multiplex assay in samples from mice. **(A)** Cytokine profiles from the prevention model. **(B)** cytokine profiles from the reversal model. Regulatory cytokines include IL-10, IL-2, IL-13, and CCL2 whereas pro-inflammatory cytokines include IFN-γ, GM-CSF, IL-1α, IL-6, IL-12, CXCL5, CXCL1, and CXCL2. Data is presented as means ± SD from 15–20 mice per group, each circle corresponds to one mouse. Statistical significance was assessed using parametric one-way ANOVA followed by Fisher’s LSD multiple comparisons test and the non-parametric Kruskal-Wallis test followed by Dunn’s multiple comparisons test (*p < 0.05, **p <.0.01).

In the prevention model, vaccine treatment significantly increased IL-10 and CCL2 while IL-2 and IL-13 remained unchanged (**Figure 3A**). The vaccine also reduced inflammatory cytokines IL-1α, IL-6, and IL-12 compared to the vehicle group. Combined vaccine and GAST-17 therapy further suppressed these inflammatory cytokines as well as IFN-γ and, GM-CSF. In contrast, GAST-17 alone increased IFN-γ, GM-CSF, IL-1α, IL-6, and IL-12. No significant changes were observed in CXCL5, CXCL1, and CXCL2 under any treatment condition (**Figure 3A**).

In the reversal model, vaccine-treated mice showed elevated levels of IL-10, IL-2, IL-13, CCL2, and IFN-γ (**Figure 3B**), with no changes in GM-CSF, IL-1α, IL-6, and IL-12 or chemokines CXCL5, CXCL1, and CXCL2, regardless of treatment (**Figure 3B**). Combined vaccine and GAST-17 therapy increased IL-2 and IFN-γ but did not affect other cytokines. Levels of IL-4, IL-5, IL-7, IL-17, and IL-1β remained unchanged (data not shown).

### Combination therapy increases regulatory T cell populations

**Figure 4:**
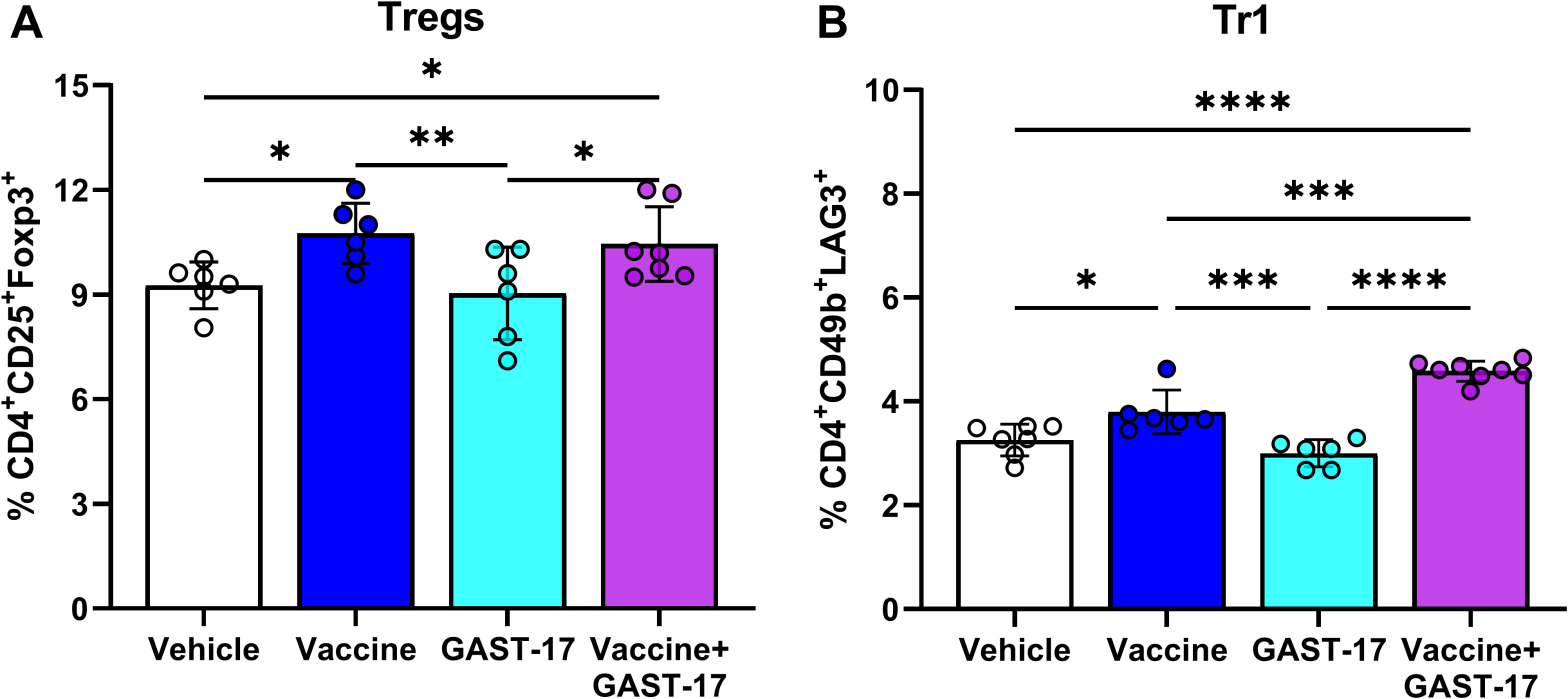
Combination therapy increases regulatory T-cells in NOD mice. Splenocytes from mice (n= 5 per group) in the indicated treatment groups were analyzed by flow cytometry. **(A)** Percentage of CD4^+^ CD25^+^ Foxp3^+^ Tregs. **(B)** Percentage of CD4^+^ CD49b^+^ LAG3^+^ Tr1 cells. Data are displayed as means ± SD from 3 independent experiments. Statistical significance was determined by parametric one-way ANOVA followed by Tukey’s multiple comparisons test (*p < 0.05, **p < 0.01, ***p < 0.001, ****p < 0.0001).

Flow cytometric analysis was performed using a sequential gating strategy to identify single live lymphocytes, followed by CD4^+^ T-cells, and CD4^+^ CD25^+^ Foxp3^+^ Tregs and CD4^+^ CD49b^+^ LAG-3^+^ Tr1 cells (**Supplementary Figure 3**). A significantly higher percentage of CD4^+^ CD25^+^ Foxp3^+^ Tregs was observed in the spleens of mice treated with the vaccine or the combined vaccine and GAST-17 therapy, compared with those receiving vehicle (Tukey’s multiple comparisons test, p = 0.02, p = 0.04) or GAST-17 alone (p = 0.008, p = 0.02) (**Figure 4A**). Similarly, the frequency of CD4^+^ CD49b^+^ LAG3^+^ Tr1 cells was elevated in mice treated with the vaccine or the combination therapy relative to vehicle-treated controls (p = 0.02, p < 0.0001) or GAST-17 alone (p = 0.0007, p < 0.0001) (**Figure 4B**). In addition, a significant increase in Tr1 cells was detected in mice receiving the combination therapy compared with vaccine alone (p = 0.0003).

### Combination therapy enhances regulatory cytokine secretion and suppresses pro-inflammatory cytokine production

To assess the functional activity of regulatory T-cells, CD4^+^ CD25^+^ Tregs and CD4^+^ CD25^–^ (including Tr1) cells were isolated from the spleens of mice treated with the vaccine and GAST-17, GAST-17 alone, vaccine alone, or vehicle. After 3 days of culture, cytokine levels in the conditioned media were determined.

**Figure 5:**
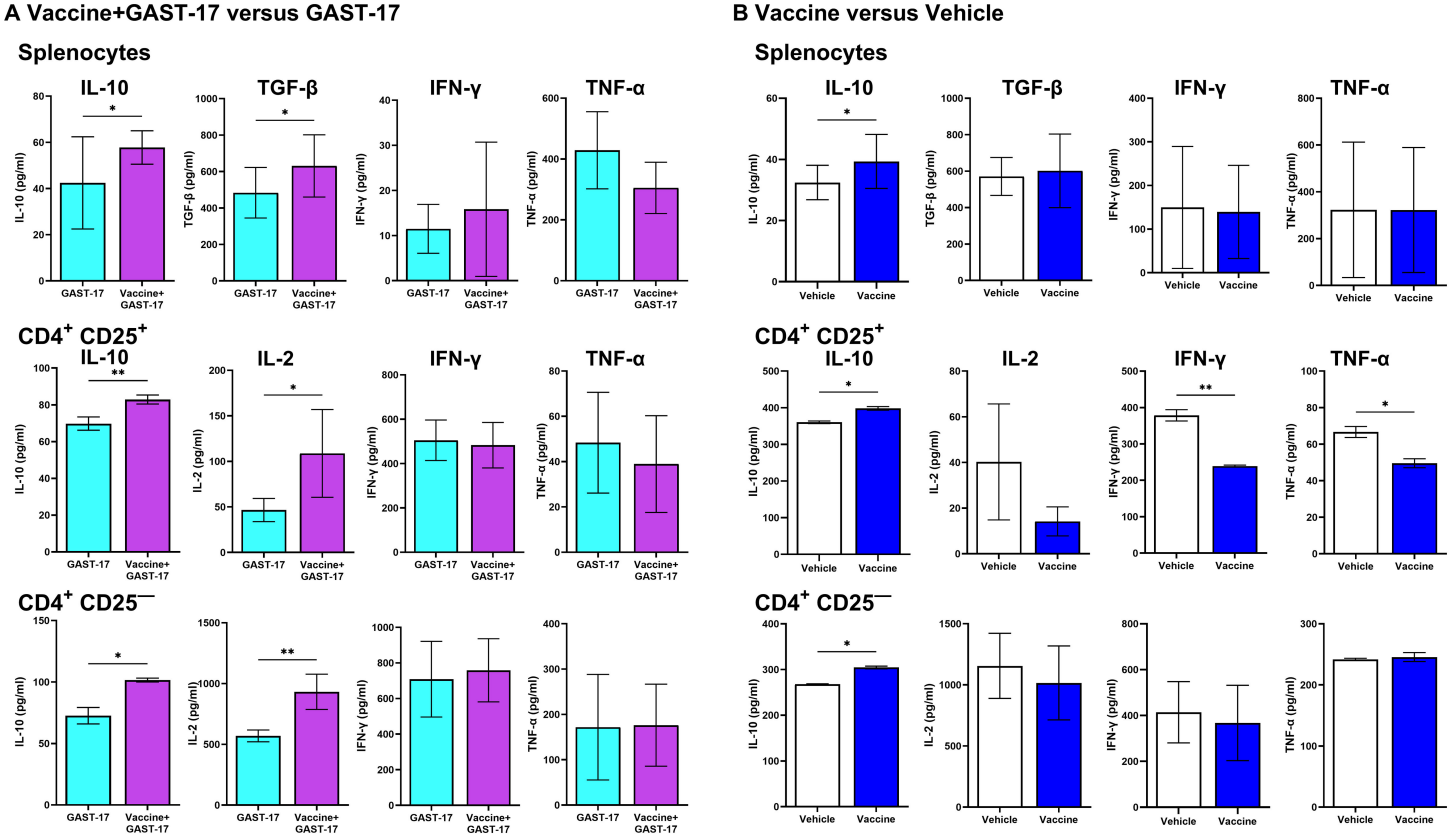
Combination therapy enhances regulatory cytokines while reducing inflammatory cytokines in T-cells. Splenocytes, CD4^+^CD25^+^ Tregs, and CD4^+^CD25^-^ cells, including Tr1 cells, were isolated from mice in the indicated treatment groups and cultured. **(A)** Cells from mice treated with vaccine and GAST-17 or GAST-17 alone. **(B)** Cells from mice treated with vaccine or vehicle. Cytokine levels of IL-10, TGF-β, IL-2, IFN-γ, and TNF-α in conditioned media were measured by ELISA. Data are presented as means ± SD from 3 independent experiments. Statistical significance was determined using unpaired parametric Welch’s t test, (*p < 0.05, **p < 0.01).

In mice receiving the combination therapy, the levels of IL-10 and IL-2 found in the media of CD4^+^ CD25^+^ Tregs (Welch’s t test, p =0.009, p = 0.01) and CD4^+^ CD25^–^ T-cells (p = 0.01, p = 0.001) were increased compared with levels found in the media of cells from GAST-17-treated mice, while IFN-γ and TNF-α levels remained unchanged (**Figure 5A**). IL-10 was significantly elevated in the media of cultured CD4^+^ CD25^+^ Tregs (p = 0.02) and CD4^+^ CD25^–^ T cells (p = 0.02), from mice given the combination treatment whereas IL-2 levels were comparable to those of vehicle controls (**Figure 5B**). Notably, IFN-γ and TNF-α levels were reduced in CD4^+^ CD25^+^ Tregs (p = 0.006, p = 0.03), but not in CD4^+^ CD25^–^ T-cells, compared with the vehicle-treated group (**Figure 5B**).

Analysis of cytokines in the conditioned media of splenocytes from mice revealed increased IL-10 and TGF-β levels from animals treated with the vaccine combined with GAST-17, and elevated IL-10 in splenocytes from mice treated with vaccine alone. IFN-γ, and TNF-α levels remained unchanged (**Figure 5**).

### *In vitro* antigen-specific stimulation of splenocytes from vaccinated mice

**Figure 6:**
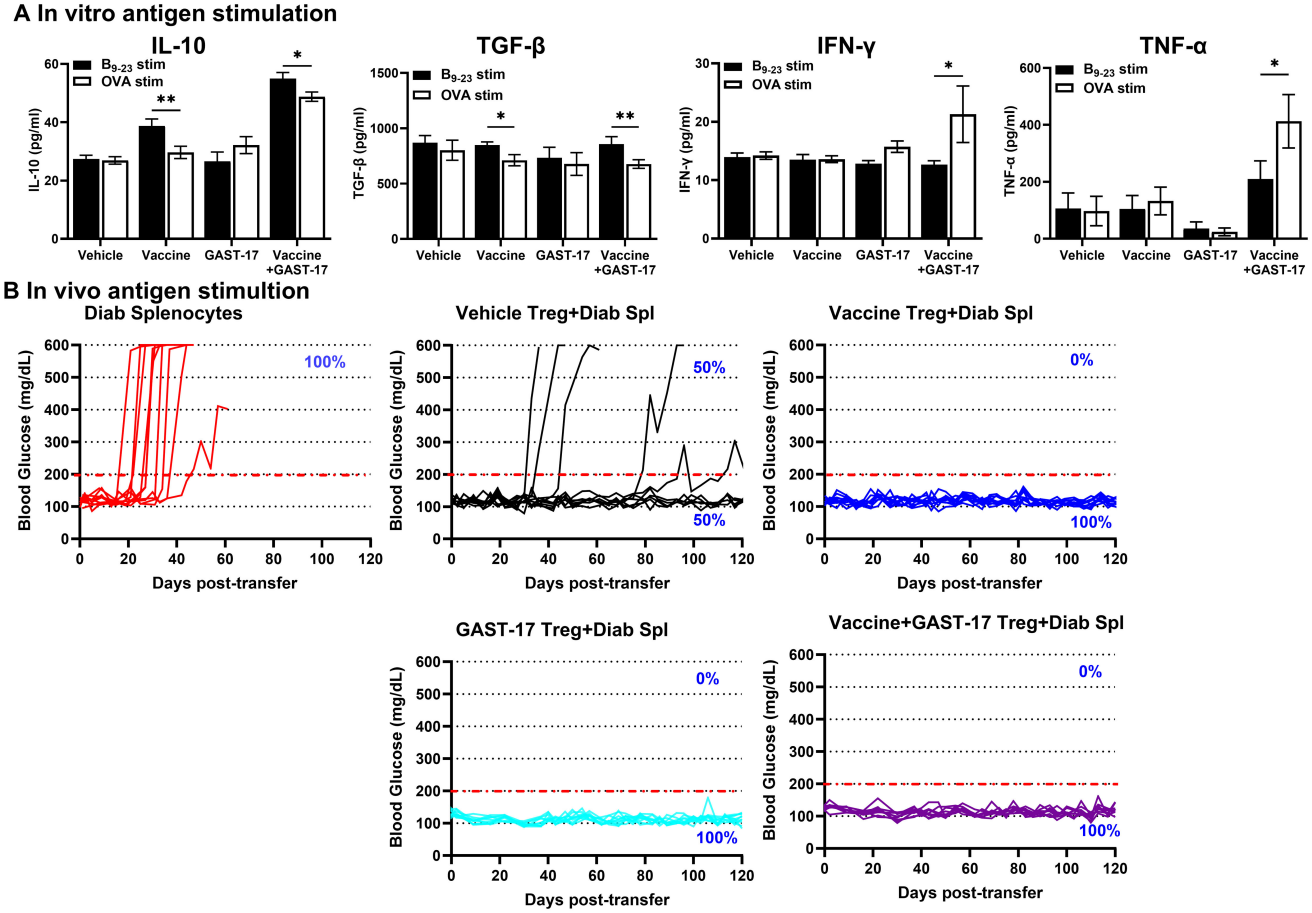
Combination therapy enhances Tregs suppressive activity. **(A)** Splenocytes from mice in the indicated treatment groups (n=5 per group) were stimulated *in vitro* with 10 µg/ml insulin B_9–23_ peptide or OVA peptide for 3 days. Cytokine levels of IL-10, TGF-β, IFN-γ, and TNF-α in the conditioned media were measured by ELISA. Data are presented as means ± SD from 3 independent experiments. Statistical significance between insulin B_9–23_ and OVA stimulation was determined by parametric two-way ANOVA (Mixed-effect analysis) followed by Bonferroni’s multiple comparisons test, (*p < 0.05, **p < 0.01). **(B)** Female 8-week-old NOD mice were orally vaccinated, and splenocyte pools (n=5 per group) were collected on day 30 post-vaccination. Diabetogenic splenocytes from diabetic NOD mice were transferred into NSG mice (n = 10 per group), either alone or together with CD4^+^CD25^+^ Tregs from mice in the indicated treatment groups. Blood glucose levels of individual mice are shown, with 200 mg/dL indicated by the red dotted line.

To evaluate antigen-specific immune responses, splenocytes from mice from each treatment group were stimulated with the insulin B_9–23_ peptide or a non-specific control peptide (OVA_323-339_) for 3 days. Analysis of conditioned media of insulin B_9–23_ stimulated splenocytes from mice treated with the vaccine or the combination of vaccine and GAST-17 revealed higher levels of the regulatory cytokine IL-10 compared with cells stimulated with the control peptide (**Figure 6A**). Stimulation with insulin B_9–23_ also increased TGF-β secretion in splenocyte cultured from mice receiving the combination therapy, but not in those treated with vaccine or GAST-17 alone (**Figure 6A**).

In contrast, IFN-γ and TNF-α levels were reduced in conditioned media from insulin B_9–23_-stimulated splenocytes of mice treated with vaccine and GAST-17, compared with OVA-stimulated controls. No significant changes IFN-γ and TNF-α were observed in splenocyte culture from mice treated with vaccine or GAST-17 alone (**Figure 6A**).

### Tregs from mice given the vaccine and GAST-17 shows *in vivo* suppressive activity

To evaluate the *in vivo* suppressive function of regulatory T-cells, an adoptive transfer study was performed. Splenocytes from diabetic NOD female mice (diabetogenic cells) were injected into immunodeficient NSG mice either alone or together with CD4^+^ CD25^+^ Tregs isolated from vehicle-, vaccine-, GAST-17-, or vaccine plus GAST-17-treated mice. Recipient mice were monitored for the development of hyperglycemia.

Transfer of diabetogenic splenocytes alone induced hyperglycemia in all recipient mice within 40–50 days post-transfer (**Figure 6B**). Co-transfer of Tregs from vehicle-treated mice delayed or prevented hyperglycemia in approximately 50% of recipients. In contrast, Tregs isolated from mice treated with the vaccine, GAST-17, or the combination therapy completely prevented hyperglycemia in all recipient mice (**Figure 6B**), demonstrating potent *in vivo* activity.

### Combination therapy increases the expression of autoimmune suppressing genes

**Figure 7:**
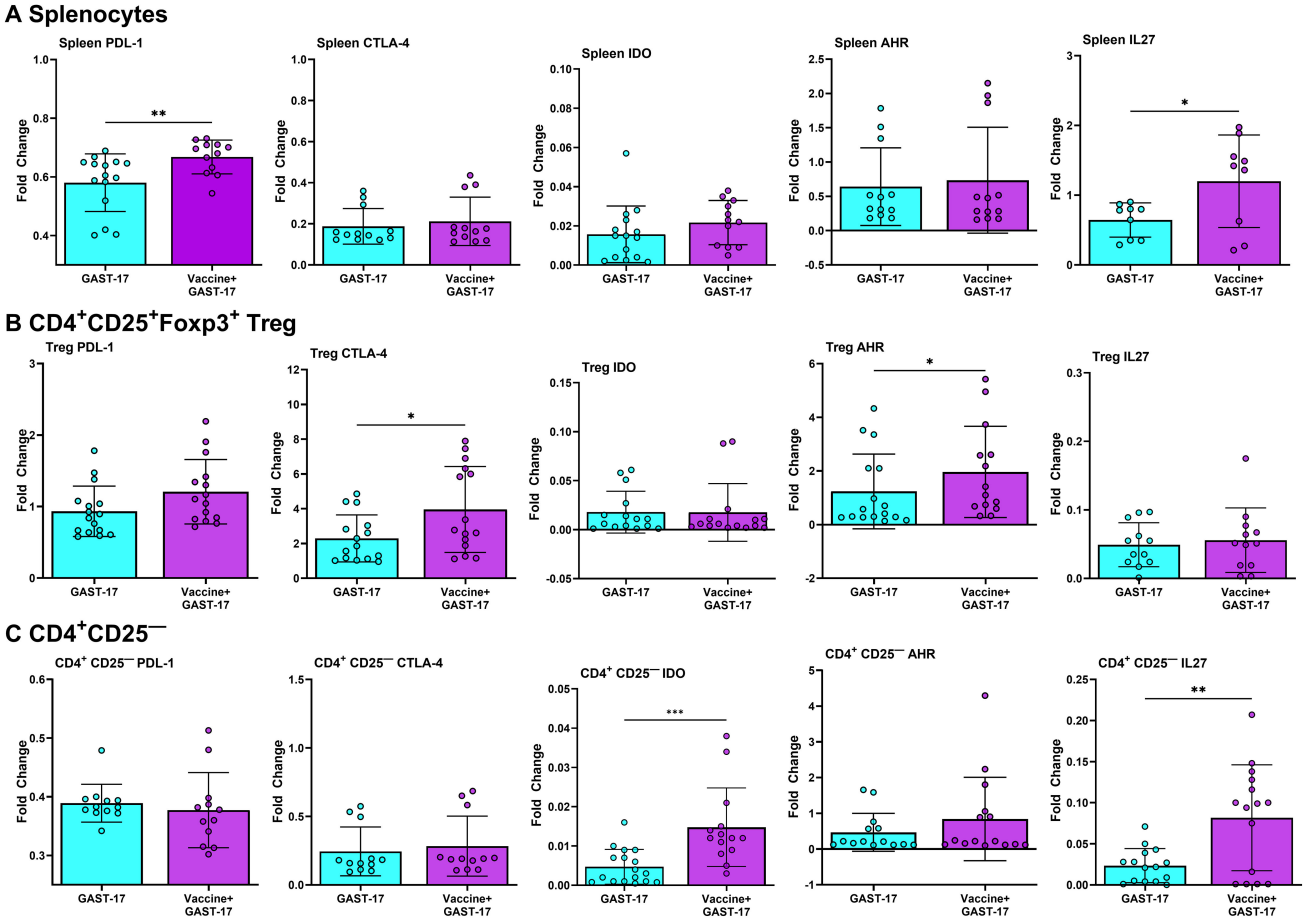
Combination therapy enhances expression of genes involved in autoimmune suppression. Thirty days post-vaccination, fold changes in PDL-1, CTLA-4, AhR, IDO, and IL27 mRNA were measured in **(A)** pooled splenocytes, **(B)** CD4^+^CD25^+^ Treg-sorted splenocytes, and **(C)** CD4^+^ CD25^–^-sorted splenocytes, from mice treated with the vaccine and GAST-17 or GAST-17 alone. Data represents mean fold changes ± SD from two independent experiments (n=6 mice per group). Statistical significance between combination therapy and GAST-17 alone was assessed using parametric Welch’s t test and the non-parametric Mann-Whitney test (*p < 0.05, **p < 0.01, *** p < 0.001).

PD-L1 and IL-27 mRNA was increased in splenocytes from mice treated with the combination of vaccine and GAST-17 (Mann-Whitney test, p = 0.006, p = 0.04) compared with those from mice treated with GAST-17 alone (**Figure 7A**). Furthermore, both splenic CD4^+^ CD25^+^ Tregs and CD4^+^ CD25^-^ T-cells from combination-treated mice showed higher expression of CTLA-4, IDO, AHR, and IL-27 mRNA relative to corresponding cells from GAST-17–treated mice (**Figures 7B, C**).

## Discussion

In this study, we evaluated a combination therapy consisting of an oral *Salmonella*-based vaccine delivering a β-cell antigen together with GAST-17 in T1D. This strategy was designed to target mechanisms contributing to T1D, including autoimmune-mediated β-cell destruction and impaired islet repair. The *Salmonella* vaccine platform has a well-established safety profile in humans and has been widely used for infectious disease prevention (29, 46–49). In preclinical studies, it effectively delivered disease-specific antigens and induced regulatory immune responses without adverse effects (17–20). GAST-17 has been reported to promote β-cell proliferation and survival in diabetic mice (39). Our study explored whether combining these interventions could improve disease outcomes, while recognizing that the mechanistic basis for such effects remains incompletely defined.

We observed that the combination therapy was associated with reduced immune cell infiltration of pancreatic islets (insulitis) and greater protection against pancreatic inflammation in both preventive and reversal models of autoimmune diabetes. Treatment with either the vaccine or GAST-17 alone also decreased immune cell infiltration of the pancreas and islets, highlighting the individual contributions of each intervention (50). These findings are consistent with prior reports that GAST-17 enhances survival and function of human islets while reducing inflammation (38, 39). Gastrin has also been associated with increase in β-cell mass and improved glucose tolerance (32, 33). Taken together, these data suggest that combining an antigen-specific vaccine with GAST-17 may help suppress autoimmunity and support β-cell function.

The *Salmonella*-based vaccine has previously been associated with expansion of regulatory T-cell populations, including Foxp3^+^ Tregs and Tr1 cells, in the spleen and lymphoid organs (18–20, 51). In our study, the addition of GAST-17 was associated with a further increase in Tr1 cell frequency, a subset that secretes IL-10 and can suppress autoreactive T-cells. Elevated IL-10 levels, together with reduced IFN-γ expression, may have contributed to a more tolerogenic immune milieu (20, 52). While these associations are consistent with regulatory activity, further studies are needed to establish direct casual links between Tr1 expansion, IL-10 production, and islet protection. Flow cytometric analyses focused on established markers used in our prior studies (CD4^+^ CD25^+^ Foxp3^+^ Tregs and Tr1 cells), allowing direct comparison with previously published datasets. While additional markers (e.g., Helios, Neuropilin-1, TIGIT, CD39/CD73) would provide a further phenotypic resolution (53–55), such analyses were beyond the scope of the present study.

Cytokine profiles and immune-regulatory gene expression also shifted following treatment, through these findings should be interpreted cautiously. Cytokine responses to antigen-specific stimulation varied across experiments, and although group level differences were detectable, their magnitude differed among individual mice. Bulk qPCR and whole-tissue cytokine measurements cannot attribute signals to specific immune cell subsets or confirm causal relationships. For example, increases in IL-10, IL-2, or IFN-γ may reflect immune activation, regulation, or compensatory responses, and the present data do not distinguish among these possibilities.

In prevention models, the combination vaccine therapy was associated with suppression of inflammatory cytokines. In the reversal models, the vaccine coincided with increased IFN−γ production, which has been reported to induce PD-L1 and other regulatory molecules that limit T-cell activity under certain conditions (56, 57). Elevated IFN−γ was accompanied by increases in IL−10, IL−2, IL−13, and CCL2, cytokines that can counterbalance inflammation and promote regulatory T-cell activity (58, 59). This cytokine signature is consistent with immune modulation observed in T1D models, the precise contribution of each cytokine remains uncertain.

Mice treated with the combination therapy also exhibited increased mRNA expression of immune checkpoint molecules CTLA-4 and PD-L1, which are known to downregulate effector T-cell responses and promote tolerance (20). The combination therapy was further associated with upregulation of the aryl hydrocarbon receptor (AhR) and indoleamine 2,3-dioxygenase (IDO), both regulators of immune homeostasis. AhR has been linked to Treg differentiation and suppression of inflammatory cytokines (60), while IDO modulate immune responses through tryptophan metabolism (61–63). IL-27 expression was also elevated consistent with its reported role in supporting Tregs induction and stability (64, 65). However, because these data were derived from bulk tissue analyses, the contributing cell types and direct functional relevance of these molecules cannot be definitively established. These findings therefore generate testable hypotheses for future mechanistic studies.

The oral *Salmonella*-based vaccine targets gut-associated lymphoid tissue (GALT), a major site for mucosal immune regulation. Attenuated *Salmonella* efficiently targets GALT-resident antigen-presenting cells, including DCs and macrophages, promoting antigen presentation that can favors tolerance (66, 67). Although prior literature supports this route of antigen delivery as a bias toward regulatory pathways, our study was not designed to define the mucosal events underlying the observed effects. Future work using cell-tracking, mucosal phenotyping, and conditional knockout models will be needed to determine whether GALT-derived responses contribute meaningfully to disease amelioration in T1D.

Re-educating the immune system to restore tolerance in individuals with T1D is an emerging therapeutic principle (26, 27). The present combination therapy reflects this approach by integrating immune modulation with β-cell support. In an ongoing trial, GAST-17 is being administered to individuals with T1D undergoing allogeneic islet transplantation. Preliminary data indicate no adverse events with GAST-17, while the number of transplanted islets required for glucose balance appeared reduced (ClinicalTrials.gov, NCT03746769) (68). These findings suggest that GAST-17 may be safe and useful in humans. Previous studies also showed that gastrin, when combined with epidermal growth factor, promoted β-cell neogenesis from exocrine ductal precursors in human islets (36, 69, 70). These observations suggest that GAST-17 may act through multiple mechanisms, although further investigation is required to clarify its precise role.

Despite these promising findings, this study has several limitations. First, the study was conducted exclusively in NOD mice, which, although widely used, do not replicate the complexity and heterogeneity of human T1D. Differences in immune regulation, disease progression, and β-cell regenerative capacity between mice and humans may limit clinical translation. In addition, tetramer-based analyses would provide valuable insight into the phenotype and functional state of PPI-specific T-cells (71, 72). However, the NOD mouse model does not express HLA-DQ8, and the use of DQ8-restricted insulin B_9–23_ tetramers would require an HLA-transgenic or humanized mouse model (73), which was beyond the scope of this study. Future investigations using HLA-transgenic or humanized models will therefore be essential. Second, the precis mechanisms underlying GAST-17-mediated effect on β-cell remain incompletely defined. Additional studies using lineage tracing, proliferation markers, and single-cell analyses will be required to distinguish β-cell proliferation, trans-differentiation, and enhanced survival. Third, cytokine and gene-expression analyses were derived from bulk tissue and stimulated splenocyte cultures, limiting mechanistic resolution and contributing to variability. Future studies employing single cell transcriptomics, adoptive transfer experiments, functional blockades, knockout models, and immune cell depletion strategies will be valuable to dissect the pathways underlying the therapeutic benefit. Finally, the study duration was insufficient to assess long-term durability or safety of the therapy, including potential effects of prolonged gastrin exposure on exocrine pancreatic tissue proliferation and cancer risk.

In conclusion, the combination of a *Salmonella*-based antigen-specific vaccine with GAST-17 was associated with reduced autoimmune pathology and improved metabolic outcomes in NOD mice. These effects coincided with induction of multiple immunoregulatory pathways and preservation of pancreatic architecture. Taken together, the findings highlight the potential of this dual-acting strategy to modulate autoimmune responses and support β-cell health, offering a promising approach that may reduce or eliminate the need for conventional immunosuppressive therapy in T1D (74).

## Data Availability

The raw data supporting the conclusions of this article will be made available by the corresponding author, without undue reservation.

## Supplementary material

Supplementary Figure 1 Combined therapy reduces hyperglycemia and reverses diabetes in NOD mice. **(A)** Kaplan–Meier plots showing the percentage of NOD mice that remained diabetes-free in prevention model. **(B)** Kaplan-Meier plots showing the percentage of mice that remained diabetic in reversal model. Differences between treatment groups and vehicle controls were evaluated using the log-rank (Mantel-Cox) test (* p < 0.05, **** p < 0.0001). Normal QQ plots were used to assess data distribution, and normality assumptions were tested using the Shapiro-Wilk test. The data were not normally distributed, as indicated by a p > 0.05. Thes results correspond to the data presented in **Figure 1**.

Supplementary Figure 2 Normal QQ plots were used to assess data distribution, and normality assumptions were tested using the Shapiro-Wilk test. The QQ plots for islet immune cells infiltration in both prevention **(A)** and reversal **(B)** models, and for insulin positive cells/islet **(C)**. The data were normally distributed, as indicated by a p < 0.05, whereas the data for insulin-positive cells were not normally distributed, as indicated by a p > 0.05. These results correspond to the data presented in **Figure 2**.

Supplementary Figure 3 Representative flow cytometry gating strategy for identification of CD4^+^ T-cell subsets. Single live lymphocytes were first gated from splenocytes, followed by gating for live CD4^+^ T-cells. The live CD4^+^ T-cell population was then further analyzed to identify two distinct subsets: CD4^+^ CD25^+^ Foxp3^+^ regulatory T-cells (Tregs) and CD4^+^ CD49b^+^ Lag3^+^ type 1 regulatory T-cells (Tr1), using matching isotype controls.
