# Supplementary material for: Oral tolerogenic vaccine combined with gastrin restores immune tolerance and beta-cell function in NOD mice with Type 1 diabetes
Source: Front Immunol. 2026 Jan 19;16:1740385. doi: 10.3389/fimmu.2025.1740385 (PMC12861915; doi:10.3389/fimmu.2025.1740385)

Oral tolerogenic vaccine combined with gastrin restores immune tolerance and beta-cell function in NOD mice with type 1 diabetes

Jacob Cobb [1], Jeffrey Rawson [1], Nelson Gonzalez [1], Fouad Kandeel [1], Mohamed I. Husseiny [1,*]

[1]Department of Translational Research & Cellular Therapeutics, Arthur Riggs Diabetes & Metabolism Research Institute, Beckman Research Institute, City of Hope National Medical Center, Duarte, California, USA.

* Corresponding: melsayed@coh.org

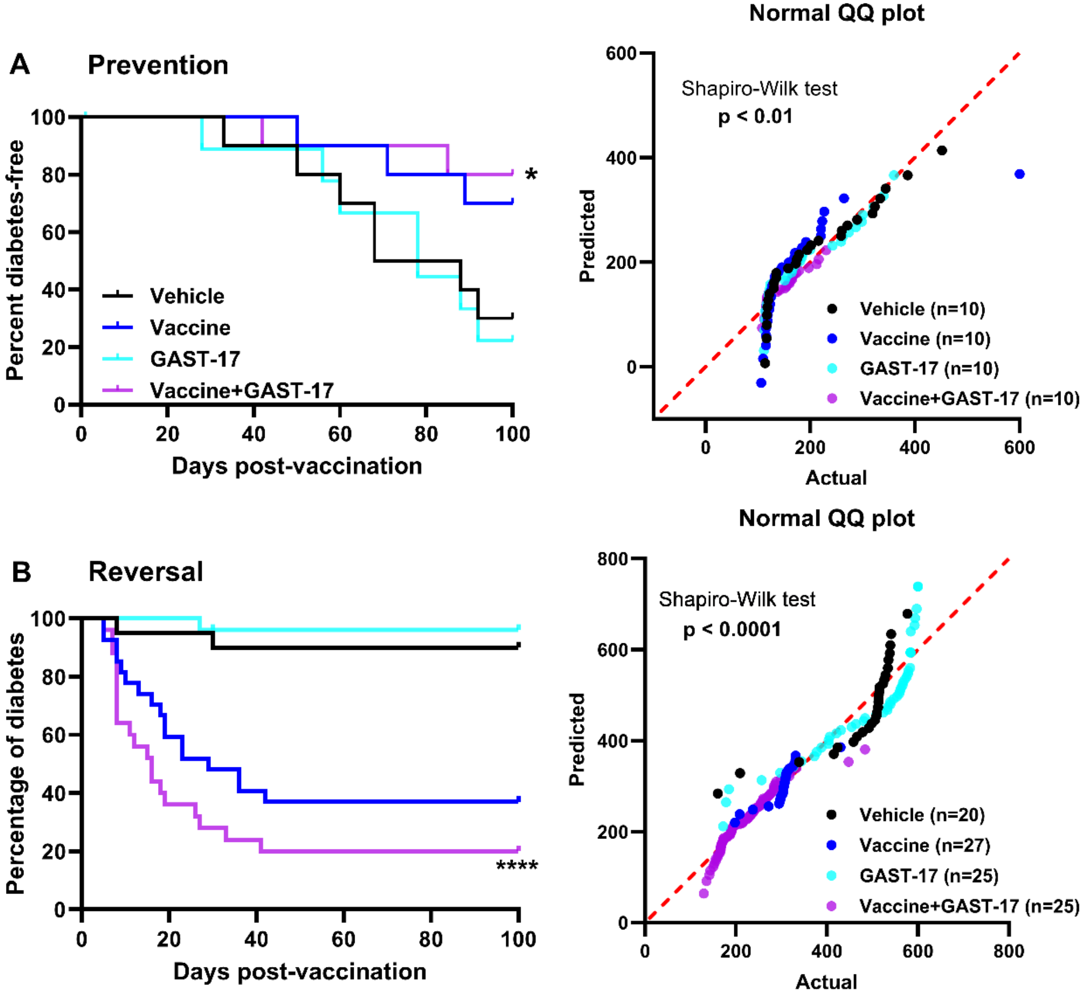

SUPPLEMENTARY FIGURE 1. Combined therapy reduces hyperglycemia and reverses diabetes in NOD mice. (A) Kaplan–Meier plots showing the percentage of NOD mice that remained diabetes-free in prevention model. **(B)** Kaplan-Meier plots showing the percentage of mice that remained diabetic in reversal model. Differences between treatment groups and vehicle controls were evaluated using the log-rank (Mantel-Cox) test (* $p < 0.05$, **** $p < 0.0001$). Normal QQ plots were used to assess data distribution, and normality assumptions were tested using the Shapiro-Wilk test. The data were not normally distributed, as indicated by a p-value more than 0.05. Thes results correspond to the data presented in **Figure 1**.

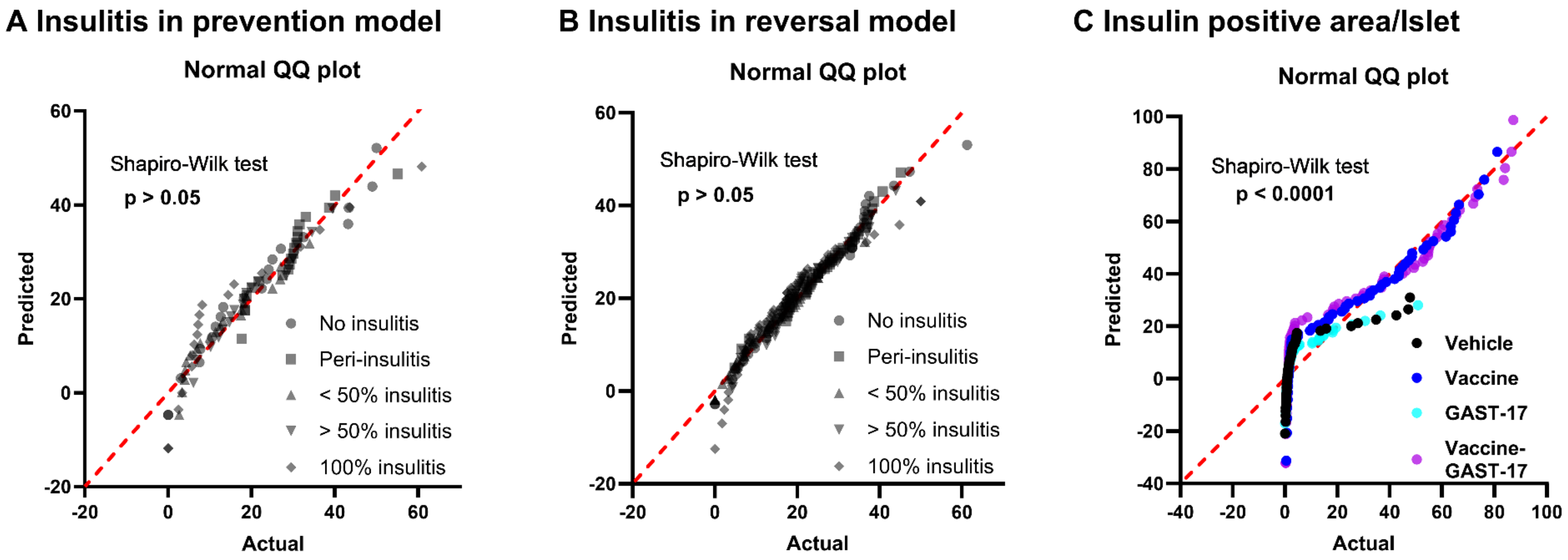

SUPPLEMENTARY FIGURE 2. Normal QQ plots were used to assess data distribution, and normality assumptions were tested using the Shapiro-Wilk test. The QQ plots for islet immune cells infiltration in both prevention **(A)** and reversal **(B)** models, and for insulin positive cells/islet **(C)**. The data were normally distributed, as indicated by a p-value less than 0.05, whereas the data for insulin-positive cells were not normally distributed, as indicated by a p-value more than 0.05. These results correspond to the data presented in **Figure 2**.

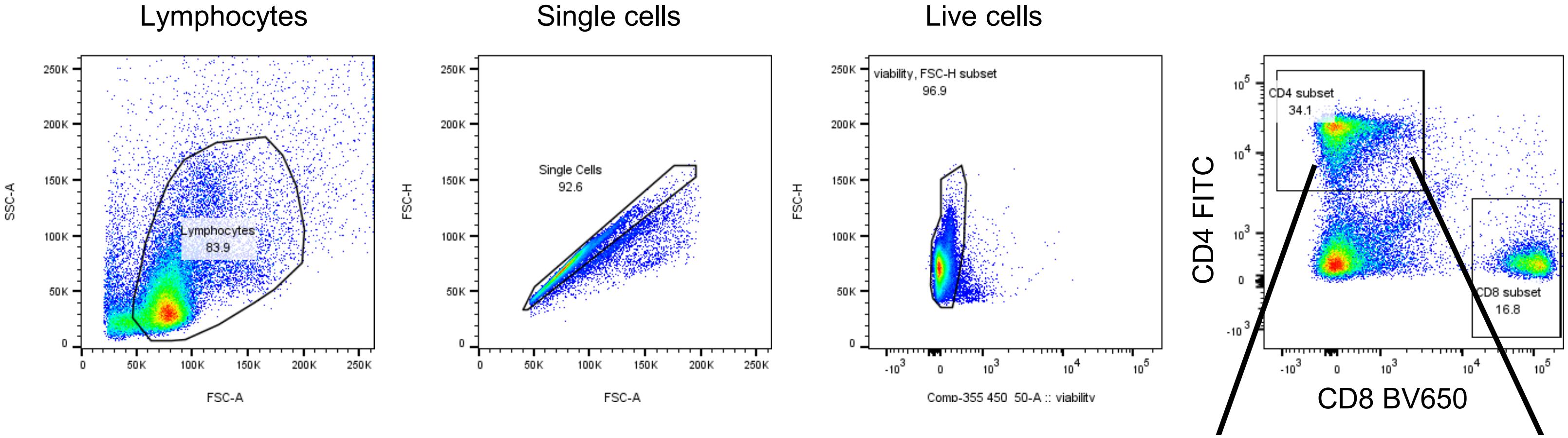

SUPPLEMENTARY FIGURE 3. Representative flow cytometry gating strategy for the identification of CD4⁺ T-cell subsets.
Single live lymphocytes were first gated from splenocytes, followed by gating for live CD4⁺ T-cells. The live CD4⁺ T-cell population was then further analyzed to identify two distinct subsets: CD4⁺ CD25⁺ Foxp3⁺ regulatory T-cells (Tregs) and CD4⁺ CD49b⁺ Lag3⁺ type 1 regulatory T-cells (Tr1), using matching isotype controls.

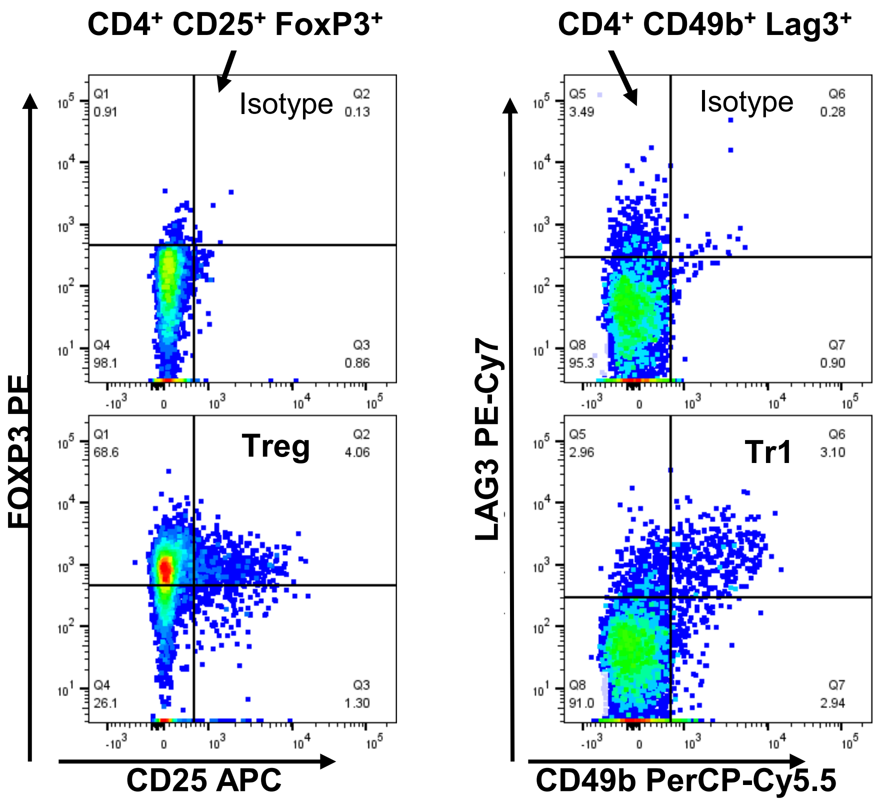

Supplement: Supplementary Figure 1 — Combined therapy reduces hyperglycemia and reverses diabetes in NOD mice. (A) Kaplan–Meier plots showing the percentage of NOD mice that remained diabetes-free in prevention model. (B) Kaplan-Meier plots showing the percentage of mice that remained diabetic in reversal model. Differences between treatment groups and vehicle controls were evaluated using the log-rank (Mantel-Cox) test (* p < 0.05, **** p < 0.0001). Normal QQ plots were used to assess data distribution, and normality assumptions were tested using the Shapiro-Wilk test. The data were not normally distributed, as indicated by a p > 0.05. Thes results correspond to the data presented in Figure 1. [file DataSheet1.pdf]
